# Fitting of Different Intraradicular Composite Posts to Oval Tooth Root Canals: A Preliminary Assessment

**DOI:** 10.3390/ma17112520

**Published:** 2024-05-23

**Authors:** Valter Fernandes, Rita Fidalgo-Pereira, Jane Edwards, Filipe Silva, Mutlu Özcan, Óscar Carvalho, Júlio C. M. Souza

**Affiliations:** 1 University Institute of Health Sciences (IUCS), Cooperativa Ensino Superior Universitário (CESPU), 4585-116 Gandra, Portugal; 2 Center for Interdisciplinary Research in Health (CIIS), Faculty of Dental Medicine (FMD), Universidade Católica Portuguesa (UCP), 3504-505 Viseu, Portugal; 3 Center for MicroElectroMechanical Systems (CMEMS-UMINHO), University of Minho, Campus Azurém, 4800-058 Guimarães, Portugal; fsamuel@dem.uminho.pt (F.S.); oscar.carvalho@dem.uminho.pt (Ó.C.); 4 LABBELS—Associate Laboratory, University of Minho, 4710-057 Braga, Portugal; 5 Clinic for Masticatory Disorders and Dental Biomaterials, Center of Dental Medicine, University of Zurich, 8032 Zurich, Switzerland; mutluozcan@hotmail.com

**Keywords:** GFRC, post, cement thickness, oval canal, microstructure, resin cement, interface

## Abstract

The purpose of the present study was to perform a preliminary analysis of the fitting of different fiber-reinforced composite (GFRC) posts to tooth root canals and determine the resin cement layer thickness. The following GFRC posts were assessed: bundle posts (Rebilda GT^TM^, VOCO, Germany), sleeve system (SAP^TM^, Angelus Ind, Brazil), and accessory posts (Reforpin^TM^, Angelus, Brazil). Twenty-four freshly extracted mandibular single-rooted pre-molars were endodontically treated and divided into six groups, according to the type of GFRC post and resin cement (self-adhesive or conventional dual-cured). Then, specimens were cross-sectioned and inspected by optical microscopy regarding the cement layer thickness and presence of defects such as pores, voids, or fissures were assessed. Bundle and accessory posts revealed a regular distribution of resin cement with a lower number of voids than found with sleeve systems. The sleeve system posts showed poor fitting at the apical portion of the root canals. The type of resin cement did not affect the thickness of the interface, although both bundle and accessory posts allow a better distribution of resin cement and fibers. The present preliminary study reveals interesting insights on the fitting of bundle and accessory posts to root dentin and resin cement layer thickness in oval-shape root canals. The sleeve system posts showed adequate fitting only at the coronal portion of the canals.

## 1. Introduction

On extensive loss of coronal tooth structure due to trauma or caries, the reconstruction of endodontically treated teeth (ETT) becomes a challenge for clinicians. The ETT maintenance is mostly influenced by factors related to the amount, design, and integrity of the remaining tooth tissue as well as the occlusal and functional loading of the patient [1,2,3]. In such conditions, the dental restoration can only be achieved using intraradicular retention with standard or custom-made synthetic posts. A wide range of ETT posts is currently available, utilizing different designs and materials. The use of cast and standard metallic posts has decreased in recent years for ETT rehabilitation due to clinical failures [4,5,6]. Thus, a mismatch in mechanical properties between metal and dentin or enamel can result in concentration of stresses at the interfaces, leading to tooth fracture [5,7,8].

The use of glass fiber-reinforced composite posts (GFRC) has been increasingly turned to by clinicians to overcome the issues with metallic posts. Auspiciously, GFRC posts reveal an elastic modulus around 18 and 45 GPa that is very close to that recorded for dentin (18–25 GPa) [9]. The match in mechanical properties provides a gradual distribution of stresses on loading. Composite posts also allow light transmittance through the materials and therefore demonstrate optical properties adequate for ETT rehabilitation. Nevertheless, the major drawback for rehabilitation with GFRC posts is related to the debonding and fractures of the resin-matrix cement which is used for intraradicular adhesion. The debonding occurs mostly due to the lack of a post’s fitting into the root canal, defects of the interface, and excessive cement thickness around the post [9,10]. The fitting and the mechanical integrity of the interface between the GFRC posts and the resin-matrix cement enhances the stress distribution through the restorative interface towards the tooth tissues [11]. Defects like pores, micro-cracks, and micro-gaps can induce stress concentrations, leading to the propagation of cracks at the interface [10]. 

The oval-shape canal is the most common root canal shape in human single-root teeth, one which has a higher prevalence, at around 53%, than that (12%) of the round shape [12,13,14]. Conventional standard GFRC posts have a round shape, and therefore they require the shaping of canals with burs. Such procedure can cause an excessive removal of remnant dentin, which negatively affects the mechanical properties of the ETT [9,15]. The root canal damage can lead to high risks of inner stress-induced fracture, since strength is directly proportional to the volume of the remaining dental structure. Previous studies clearly show that a high volume of dentin assists the proper mechanical behavior of the remaining tooth structure [15,16,17,18,19]. From a mechanical perspective, the post-space preparation should be restricted to solely cleaning the canal walls by removing the smear layer and any potential remnant filling materials. It should be emphasized that further removal of dentin must be avoided on accomplishing the endodontic treatment [18]. Accordingly, novel GFRC designs and materials have been developed, such as bundle and sleeve system posts. Bundle GFRC posts are composed of several fine individual posts 0.3 mm in diameter. On fitting, the bundles spread into the root canal space, adapting to any root canal shape [1,4,20,21]. The sleeve-system GFRC post (e.g., SAP^TM^, Angelus, Brazil) comprises a single drill, post, and sleeve, offering a viable alternative to the conventional extensive inventory of diverse drill and post models. The manufacturer has claimed that the sleeve system ensures proper fitting and mechanical retention in tooth root canals and that they can be used in different diameters [19,22,23]. Other studies have indicated that the placement of accessory posts with smaller dimensions can decrease the number of catastrophic fractures, specifically reducing those involving the middle or apical third of the root [24,25,26,27]. Thus, the filling of canal space with a higher percentage of fibers allows a better fitting of the posts and therefore decreases the resin cement thickness. 

In view of the previous scientific debate, the aim of the present study was to conduct a preliminary in vitro assessment of the fitting of different GFRC posts into root canals and the resultant resin cement layer. The null hypothesis was that the different GFRC post design can provide a similar fitting and level of presence of defects at the adhesive interface.

## 2. Materials and Methods

### 2.1. Preparation of Specimens

**Figure 1:**
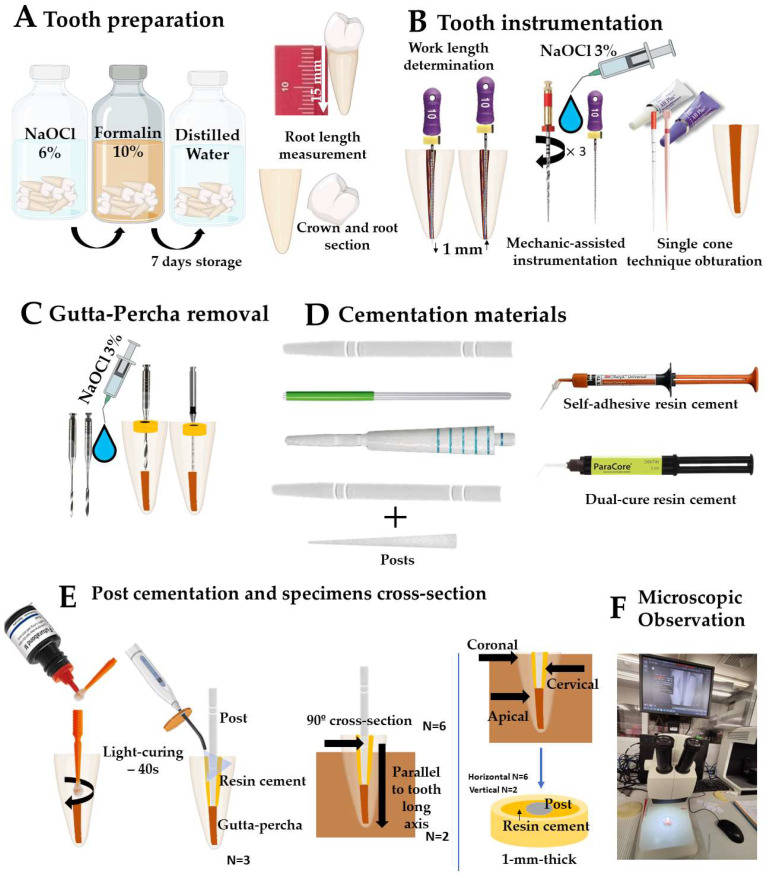
Schematics of the preparation of specimens. (**A**) Tooth preparation. (**B**) Tooth instrumentation using WaveOne^TM^ primary # 25.08 files, 25 mm (Dentsply-Maillefer, Ballaigues, Switzerland). (**C**) Gutta-percha removal. (**D**,**E**) Cementation of GFRC posts, self-adhesive resin cement (SA) and dual-cured resin cement (CD). (**E**) Cross-sectioning at three different regions, coronal, middle, and apical, and then (**F**) preparation for microscopic observation.

The present study was formerly examined and accepted by an institutional reviewing board at the University Institute of Health Sciences (IUCS), CESPU, Portugal, with the following ethics protocols reference: CE/IUCS/CESPU-18/2022. All procedures carried out including human participants are performed in accordance with the ethics standards of the IUCS ethics committee, and compliant with the 1964 Helsinki declaration and its later adjustments or analogous Ethics Standards. Informed consent was pointless, taking into account the national regulations and since all data were anonymous. The prerequisite for informed consent was also waived by the ethics committee/Institutional Review Board of IUCS at CESPU, Portugal. Twenty-four single-root human mandibular premolar teeth (mean root length of 15 mm) with totally formed apexes were chosen for this study, taking into account root sizes and absence of caries, noticeable fracture lines, or cracks [28]. Tooth assessment was carried out regarding the root canal diameter and shape. Also, only teeth with large and oval root canals were chosen. Teeth were earlier extracted due to orthodontic and periodontal reasons at the IUCS, CESPU. After extraction, teeth were instantly immersed in 6% sodium hypochlorite solution (NaOCl) (CanalPro^TM^, Coltene/Whaledent Altstätten, Switzerland) for 5 min. Afterwards, teeth were immersed in 5% formalin at room temperature for 7 days and then immersed in distilled water for 7 days. The anatomic crowns were firstly cross-sectioned, and all teeth were endodontically shaped. The working length was determined by using an endodontic file type K-flexofile ISO # 10 until it was visible through the apical foramen, and then 1 mm was withdrawn. Mechanically-assisted shaping was performed using reciprocating friction drives (25 mm in length) with # 25.08 primary files (Wave One^TM^, Dentsply-Maillefer, Ballaigues, Switzerland), as shown in Figure 1A.

**Figure 2:**
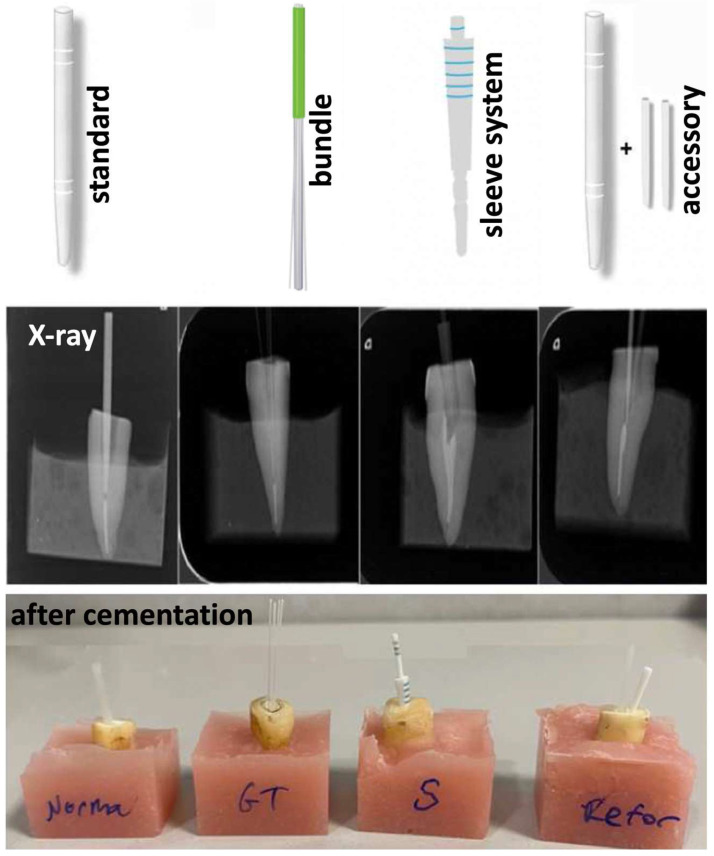
Schematics of GFRC post types (standard, bundle, sleeve, and accessory) after X-ray and cementation.

The root canal teeth were disinfected using 3% NaOCl (CanalPro^TM^, Coltene/Whaledent Altstätten, Switzerland) on each filing, on which a permeabilization procedure was performed with a 10K file between every 3 reciprocating drives, using a syringe with a lateral irrigation needle (30G) (Figure 1B). Tooth root canals were dried with calibrated paper cones (Dentsply-Maillefer, Ballaigues, Switzerland). Finally, root canals were filled using calibrated primary gutta-percha cones (Dentsply Maillefer, Switzerland), plus single cone technique and vertical compaction with gutta-percha points, and finally embedded within resin-matrix cement (AH-Plus^TM^, Dentsply-Maillefer, Ballaigues, Switzerland) [29]. Then, the tooth root canal space was shaped using reamers sized 2, 3, and 4 (Largo Peeso reamers ^TM^, Dentsply-Maillefer, Ballaigues, Switzerland). The intraradicular space shaping was accomplished by removing 10 mm gutta-percha from the canal using a 1.5 mm ∅ post bur (Parapost nº 6 Black P-42^TM^, Coltene/Whaledent, Cuyahoga Falls, OH, USA) at an 800-rpm speed (Figure 1C). Excessive pressure of instruments against the intraradicular dentin surfaces when using either Largo reamers or drills was avoided. Silicone stops (Dentsply Intl, Charlotte, NC, USA) were placed on each drill to ensure that the tooth root canal shaping was achieved at the beforehand-settled lengths. The debris released after each drilling was rinsed away with 2 mL of 3% NaOCl. Tooth root canals were thoroughly dried with paper points after shaping. X-ray images of tooth roots were attained using an X-ray clinical apparatus (Corix 70 Plus KVP X-ray^TM^, CORAMEX S.A, Mexico City, Mexico) to examine the gutta-percha removal (Figure 2). X-ray analyses were acquired using a triangular scanning technique at 70 kVp and 8 mA for 53 s. Twenty-four prepared roots were then randomly separated into four experimental groups according to the sort of GFRC post, and then into two subgroups based on the type of resin cement (Figure 1D).

Before cementation, the root canals were washed with 96% ethanol, and then dried with paper points [15]. The following GFRC posts were assessed: standard GFRC posts (Rebilda^TM^, VOCO, Cuxhaven, Germany), bundle posts (Rebilda GT^TM^, VOCO, Cuxhaven, Germany), sleeve systems (SAP^TM^, Angelus, Londrina, Brazil), and accessory posts (Reforpin^TM^, Angelus, Londrina, Brazil). The data on the GFRC posts is shown in Table 1. At first, GFRC posts were placed into each canal and the fitting was evaluated by X-ray analyses (Figure 2). After checking the correct positioning of each post, GFRC posts were removed from each canal and disinfected once again with ethanol.

The cementation procedure was performed with a self-adhesive (Rely X U200^TM^, 3M, Maplewood, MN, USA) or conventional dual-cure resin cement (ParaCore Automix^TM^, Coltene Whaledent, Cuyahoga Falls, OH, USA). On conventional cementation, intraradicular dentin was previously conditioned using a universal adhesive system (Parabond adhesive^TM^, Coltene Whaledent, USA) according to the manufacturer’s instructions. The universal bonding agent was applied inside the root canals using a fine microbrush with reciprocating friction movement for 30 s (Figure 1E). Following this, the resin-matrix cement material (ParaCore Automix^TM^, Coltene Whaledent, USA) was applied directly into the intracanal space using a syringe tip. On self-adhesive cementation, the resin-matrix cement material (Rely X U200^TM^) was applied directly into the intracanal space, using a syringe tip, in a one-step technique. The GFRC post was also coated with the cement and then placed into the tooth root canal using slight pressure. The dental inspector apparatus (Ney surveyor^TM^, Hanau, Germany) was used to align the post space with the long axis of the tooth. The excessive cement layer was removed, and the cement was then light-cured using a light curing unit (LCU) with irradiance at 800 mW/cm^2^ and wavelength at 420–480 nm (LY-A180^TM^, Anyang Zongyan Dental Material Co., Anyang, China) for 40 s (Figure 1E). After cementation, periapical X-ray analyses were performed for evaluation of fitting (Figure 2). Specimens were then assembled with a self-curing polyether modified resin (Technovit 400^TM^, Kulzer GmbH, Wasserburg, Germany) in a short length of polyvinyl chloride mold (Figure 2) [30,31,32,33]. All the specimens were maintained in 100% humidity at 37 °C for 24 h. Specimens were cross-sectioned at longitudinal and transversal planes relative to the long axis of the GFRC posts for inspection of the resin-matrix cement layer, as shown in Figure 1E. Groups of specimens were cross-sectioned at three different regions (cervical, coronal, and apical). Cross-sections were performed, resulting in slices with 1 mm thickness, using a precision cutting-machine (Isomet^TM^, Buhler, IL, USA).

### 2.2. Microscopic Analysis

Cross-sectioned specimens of each group were ultrasonically cleansed using deionized water for 10 min, then immersed in 96% ethanol for 2 min, and then air-dried. Cross-sectioned specimens were inspected by optical microscopy at magnifications ranging from ×100 up to ×1000. Microstructural analyses were performed using an optical microscope (Leica DM 2500 M™; Leica Microsystems, Wetzlar, Germany) connected to a computer for image processing, using Leica Application Suite™ software program v 41 (Leica Microsystems, Wetzlar, Germany). A quantity of six micrographs were acquired at ×350 magnification for each specimen (*n* = 18). Black and white images of the interface were evaluated using the Adobe Photoshop^TM^ software program (Adobe Systems software program, v23.5.5 Dublin, Ireland), with the black regions representing the post or voids/pores in the cement while the white regions represented the resin cement and dentin. The cement layer thickness on the cross-sections and the porosity were evaluated using the Image J^TM^ software program v154 (National Institutes of Health, Bethesda, MD, USA). Measurements of cement thickness on mesial, distal, vestibular, and lingual were carried out at each horizontal cross-section. On the vertical cross section, measurements were performed on mesial and distal, apical, medial, and coronal parts to access the mean distance and standard deviation between the GFRC post and root dentin. In this study, the normal distribution of the data was evaluated applying the Kolmogorov–Smirnov test followed by the Levene test, in which the *p*-value was set for a significance level of 5% (<0.05). Student’s *t*-test for independent samples was used for analysis. All statistical tests were performed using SPSS software program v28.0.0 (SPSS, Chicago, IL, USA). A significance level of 5% was adopted.

## 3. Results

**Figure 3:**
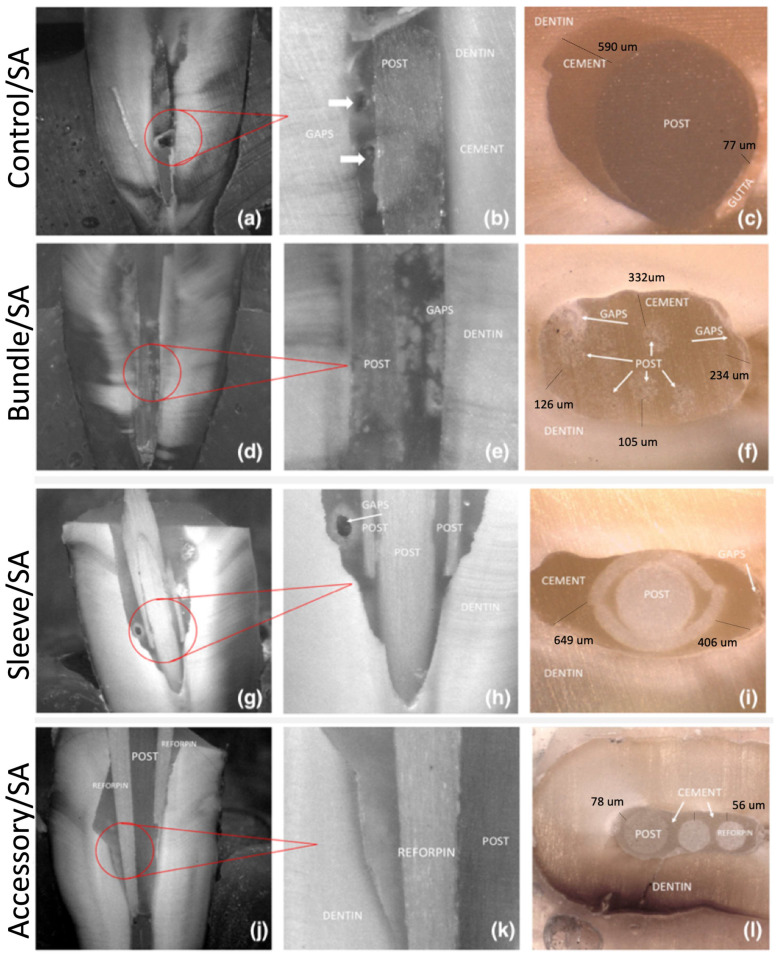
Optical microscopy images of the interfaces after cross-sectioning GFRC posts to resin-matrix cement and tooth for the groups with self-adhesive (SA) conditioning: A1 control/SE group (**a**–**c**); B1 bundle/SE group (**d**–**f**); C1 Sleeve (SAP)/SE (**g**–**i**); D1 accessory/SE (**j**–**l**). Vertical section at ×10 and ×25 and horizontal section at ×35.

**Figure 4:**
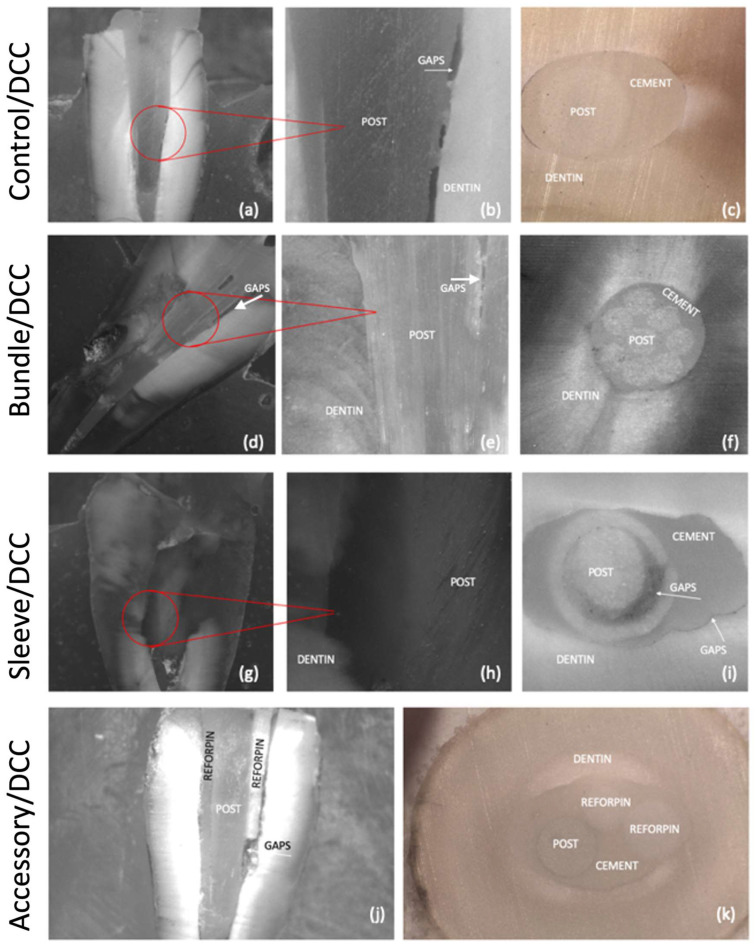
Optical microscopy images of the interfaces after cross-sectioning GFRC posts to resin-matrix cement and tooth for the groups with traditional dual-cured (DCC) resin cements: A2 control/DCC group (**a**–**c**); B2 bundle/DCC group (**d**–**f**); C2 Sleeve (SAP)/DCC (**g**–**i**); D2 accessory/DCC (**j**,**k**). Vertical section at ×10 and ×25 and horizontal section at ×35.

Optical microscopy images of the adhesive interfaces of the GFRC posts are shown in Figure 3b and Figure 4. Regarding the control group (A1), the presence of gaps and voids can be noted in Figure 3a,b and Figure 4a,b, along with an imbalanced distribution of the resin cement between the GFRC post and the dentin substrate (Figure 3c and Figure 4c).

**Figure 5:**
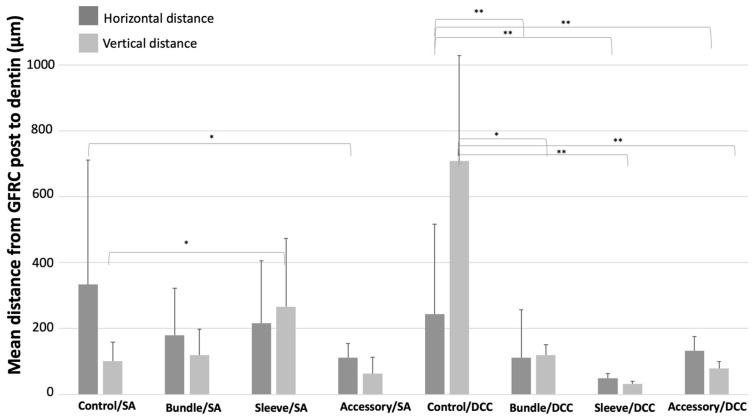
Mean distance and standard deviation from post to dentin, measured in horizontal and vertical cross sections at ×35 magnification. * Statistically different < 0.05; ** Statistically different < 0.005.

On the bundle groups (B1 and B2), some gaps with smaller size were detected, which were predominantly located at the cervical level (Figure 3d). The resin cement was properly distributed in the GFRC posts occupying a regular intraradicular space (Figure 3 and Figure 4). Specimens from the sleeve groups (C1 and C2) revealed the highest size of gaps, as seen in Figure 4g and Figure 5 (*p* < 0.05). Also, an additional shaping of the cervical portion of the tooth canal was required previous to the cementation of the sleeve-system GFRC post. This decreased the amount of tooth structure.

A well-distributed layer of resin cement can be noted around the accessory GFRC posts, along with a few gaps (Figure 4j,k). The lowest mean distance between GFRC posts and dentin can be seen in Figure 3d, Figure 4d and Figure 5 (*p* < 0.05). The secondary posts occupy space in the canal, reducing the amount of cement between the posts and the dentin. 

The highest mean distance values were recorded for the control group after cementation with self-adhesive or traditional resin cement (*p* < 0.05). On self-adhesive cementation, the lowest distance between GFRC and dentin was recorded for the groups with accessory GFRC posts. On conventional cementation, the lowest distance between GFRC and dentin was recorded for the groups with sleeve-system GFRC posts.

## 4. Discussion

In this pilot study, four different types of glass fiber-reinforced posts (GFRC) cemented with two types of resin cements in shaped root canals were assessed. Thus, the preliminary findings reported in this research revealed that the use of different GFRCs with different designs influences the thickness and the adhesive interface of the resin cement. Thus, the present findings rejected the null hypothesis in this study since different GFRC post design resulted in variable presence of voids or gaps in flared canals. According to the present findings, the resin cement layer thickness decreases with the proper fitting of GFRC posts and therefore an improved distribution of the cement layer is noticed with a lower number of defects, such as gaps and voids, which is in accordance with other previous studies in literature [34,35]. 

An irregular distribution of resin cement layer thickness was noticed around the standard GFRC post (control group), with mean values ranging from 9 up to 690 μm. Such results were also found in other studies on the influence of a higher thickness of resin cement, and the consequent increase of structural defects, volumetric shrinkage, and polymerization stress [35,36]. Voids, pores, micro-cracks, and micro-gaps were found at the cement-to-root-dentin interfaces, resulting in spots for stress concentration, leading to crack propagation and catastrophic fractures [35,36]. Other studies have shown opposite results and validate the influence of the layer thickness on the retention of the intracanal posts [37]. However, a thick layer of resin cement is susceptible to air bubbles and voids, which would increase the chances of failure and displacement of the GFRC post [10]. The clinical success of GFRC posts significantly depends on the fitting and frictional retention to the intraradicular dentin [38]. Thus, bundle GFRC post systems filled the canal space, as demonstrated by optical microscopy images, leading to a higher contact area to the intraradicular dentin surfaces. 

The distribution of several thin GFRC posts (0.3 μm in diameter) in the tooth root canal space allows a reduced cement thickness with a low number of defects. The GFRC post can also optimally adapt to different root canal design such as curved, oval, or conical root canals [20,25,39]. Previous studies on mechanical behavior have shown that bundle GFRC posts exhibit higher bond strength compared to other systems [27,39]. Also, the number of thin filaments at the coronal portion enhances fitting and bonding to the intraradicular dentin surfaces, resulting in a homogenous stress distribution compared to standard GFRC posts [25,26,40]. Our results partially confirm these findings, since bundle posts have the smallest average resin cement thickness, in both vertical and horizontal cross-sections, which is in accordance with other studies in literature [20,41]. Nevertheless, some studies have reported lower bond strength values for bundle GFRC posts at the cervical region, due to wider space among the filaments [40,42]. The use of bundle GFRC posts can reveal challenges regarding the cementation, given the removal of the plastic holder of the filaments and the consequent formation of bubbles in the resin cement after placement [1]. Thus, the addition of accessory thinner posts can fill spaces among bundle GFRC posts and increase the overall fitting [20,41]. The present results show a homogenous distribution of resin cement, with the lower mean values of distance between the filaments and dentin. Studies have evaluated the effectiveness of accessory GFRC posts in restoring endodontically treated teeth and compared it to other root reinforcement approaches [25,27]. Previous results revealed that accessory GFRC posts could also be used as an alternative to the resin composites. A recent study showed the adequate mechanical properties of filled and unfilled hollow GFRC posts. Such filled posts have better mechanical properties than the standard GFRC posts included in our study. Also, this technique allows the use of posts as carriers and simplifies the operative protocol [41].

Large gaps and resin cement thickness were recorded for sleeve GFRC systems due to the misfit between the sleeve and dentin surfaces, which also implies a need for further shaping of the cervical portion of the canal. Also, the sleeve GFRC system provided an imbalanced distribution between the canal and the GFRC post. Two articles assessed the push-out bond strength of different post systems, including the sleeve GFRC system [22,39]. The group that comprised sleeve systems showed the smallest values of dentin thickness at the coronal portion. An adequate bond strength was reported when combining a dual-cured adhesive system and the sleeve GFRC system [39]. Another study revealed a high bond strength of the sleeve system and the custom-made GFRC posts to dentin. The results of the previous study revealed that both post systems exhibited high bond strength values to dentin [22]. However, there are limited number of studies on the sleeve system, which does not allow for extensive comparisons with other studies. In fact, the analysis of the GFRC fitting is essential to ensure the mechanical stability of endodontically treated teeth restorations.

Our results are in accordance with this study, considering they show an equal distribution of cement, with the lower mean values of distance between the fibers and dentin. A recent article tested the mechanical properties of filled and unfilled hollow posts; it was concluded that both filled and unfilled hollow posts have good mechanical properties; however, filled posts demonstrated better performance. These posts are more resistant than GFRC posts, like the A1/A2 posts included in our study, indicating that the hollow posts are more resistant. Also, this technique allows the use of posts as carriers, and simplifies the operative protocol [41,43].

Regarding the type of resin cement used on GFRC post cementation, the different sections do not show any differences in distribution between the post and dentin after cementation with self-adhesive resin cements or dual-cured resin cements. Defects found in the GFRC posts occurred due to the design of the intraradicular space [14,44]. The most common commercially available resin cements often used by clinicians are dual-cured and self-adhesive resin cements, as used in our study as a cementation material [45,46]. The two resin cements have similar chemical compositions; the inorganic components frequently comprise micro and/or nano-scale colloidal silica, barium silicate, or zirconium silicate particles. The organic matrix often involves methacrylate-based monomers (i.e., BisGMA, UDMA, TEGDMA) and photoinitiators, although the self-adhesive resin cement includes 10-MDP or 4-META acidic monomers that evince self-adhesive properties with respect to tooth tissues [47,48,49]. Dual-cured resin cements require multi-step adhesive procedures, with prior conditioning with an etch-and-rinse or self-etch (SE) adhesive [50,51]. However, the contacts between root dentin and acidic adhesive monomers can be impaired by the thick amounts of smear layer, therefore decreasing the mechanical interlocking of the resin cement. The bond strengths of dual-cured resin cements are higher than those recorded for self-adhesive resin cements [52]. Other studies have demonstrated that self-adhesive resin cements have similar clinical performance compared to dual-cured resin cements [47,53,54,55,56,57,58,59]. Also, some studies showed that self-adhesive cements have limited capacity to diffuse and decalcify the underlying dentin [60,61,62,63,64]. That can be attributed to the chemical interaction between the acidic monomers in the resin cement and the hydroxyapatite. 

After endodontic treatment, the mechanical properties of the remnant tooth tissues can decrease, regardless of the use of an irrigant solution such NaOCl and ethylenediaminetetraacetic acid (EDTA) [53,56,57,58,59].

## 5. Conclusions

The major conclusion deals with the adequate spatial distribution of resin cement and glass fiber-reinforced composite posts when the bundle and accessory posts were combined within a multi-filament system. In fact, the fitting of the glass fiber-reinforced posts into the intraradicular was improved, which resulted in less space among the filaments, which were filled with the resin cement. On the other hand, the sleeve system produced an increase in the number of gaps, in the intraradicular space. The type of resin cement used does not have an impact on the occurrence of gap formation around the dental post. Further studies are required to address the issues regarding the fitting and distribution of resin cements among glass fiber-reinforced composite posts.

## Figures and Tables

**Table 1:** Materials and groups assessed in the present study.

Group; Material (Brand, Manufacturer, Country)	Organic Matrix (wt.%)	Inorganic Fillers (wt.%)	Filler Shape and Type	Elastic Modulus and Fracture Load
Control group, Standard GFRC post (Rebilda^TM^, VOCO, Cuxhaven, Germany)	UDMA; DMA (20 wt.%) [1]	70–80	Glass fibers (10–20 μm) with SiO_2_, SnO_2_, B_2_O_3_, Al_2_O_3_ alkali oxides Coronal diameter: 2 mm Apical diameter: 1.02 1.5 mm diameter	*E*: 18–30 GPa; *F*: 400–600 N
Bundle GFRC post (Rebilda GT^TM^, VOCO, Cuxhaven, Germany)	UDMA; DMA (20 wt.%)	70–80 [1]	Glass fibers (10–20 μm) with SiO_2_, SnO_2_, B_2_O_3_, Al_2_O_3_ alkali oxides 12 single narrow GFRC filaments 1.4 mm diameter Single GFRC filament diameter: 0.3 mm [2]	*E*: 31.5 GPa; *F:* 1040 N
Sleeve-system GFRC (Splendor SAP^TM^, Angelus, Londrina, Brazil)	Epoxy resin (19–20 wt.%)	50–80	Glass fibers, type E or E-glass, with SiO_2_ (55–65%),CaO (9–25%), B_2_O_3_, Al_2_O_3_ (15–30%) alkali oxide metals ^a^ [3] Main Post Diameter: 1.0 mm Sleeve Diameter: 1.4 mm Sleeve taper: 0.8 mm	*E*: 37 GPa [4]; *F*: 835.9 N
Accessory GFRC posts (Reforpin^TM^, Angelus, Londrina, Brazil)	Epoxy resin (19–20 wt.%)	80	Glass fibers, type E or E-glass, with SiO_2_ (55–65%),CaO (9–25%), B_2_O_3_,Al_2_O_3_ (15–30%) alkali oxides metals ^a^ [3] Diameter: 1.3 mm Length: 14 mm	*E*: 35–45 GPa; *F*: 569.5 N
Self-adhesive resin cement (RelyX U200^TM^, 3M, Maplewood, MN, USA)	TEGDMA, 3-propanediyl dimethacrylate and phosphorus oxid, propyl and phenyltrimethoxy silane, propenoic acid, 2-methyl-, 2-hydroxy-1, 3-propanediyl dimethacrylate and phosphorus oxide and phosphoric acid groups (28 wt.%) [5,6]	72 [5]	Trisilane-treated silica, powdered glass, chemical glass oxides (non-fibrous), glass fillers, glass fibers, acetic acid, copper sodium monohydrate [7]	*E*: 15.99 GPa; *FS*: 81.29 MPa [8]; VMH: 58.64 HV (cervical) and 56.1 HV (apical) [9]
Group CDD, Dual-cured resin cement (Paracore^TM^, Coltene Whaledent, Cuyahoga Falls, OH, USA)	Bis-GMA, UDMA, TEGDMA, DDDMA, TMPTMA, BHT, dibenzyl peroxide, CQ, accelerators [10]	74 [11]	Amorphous silica, zinc oxide, barium glass, and sodium fluoride particles Particle size: 0.01–5 μm [10,12]	*E*: 9.2 GPa [10]; *FS:* 120 MPa [11] *BS*: 280 MPa; VMH, 43.2 HV [13]
(Parabond^TM^, Coltene Whaledent, Cuyahoga Falls, OH, USA)	Adhesive A: Methacrylate (HEMA) (39%), Maleic acid (1.5%), Benzoyl peroxide (1.5%). Adhesive B: Ethanol (80%), Water and Initiators. [14,15] PH: 0.9–1.3 [16]	52	Micro-scale barium glass ceramic and zirconium glass ceramic micro-scale particles at 1 μm Nano-scale SiO_2_ particles at around 20–40 nm [16]	*E*: -; FS: -; SBS: 5.44 MPa

Triethylene glycol dimethacrylate (TEGDMA); N,N-Dimethylaminoethyl acrylate (DMA); Bisphenol A-glycidyl dimethacrylate (Bis-GMA); Urethane dimethacrylate (UDMA); Camphorquinone (CQ); Dodecylmethacrylate (DDMA); 2-hydroxyethyl methacrylate (HEMA); Butyl hydroxytoluene (BHT); Silicon oxide (SiO_2_); Trimethylolpropane trimethacrylate (TMPTMA); Vickers microhardness (VMH); Flexural strength (*FS*); Maximum flexural force (*F*); Shear bond strength (*SBS*); Elastic modulus (*E*).

## Data Availability

Data are contained within the article.
